# 3′,7′,7′-Trimethyl-1′-phenyl-5′,6′,7′,8′-tetra­hydro­spiro­[indoline-3,4′-(1*H*,4*H*-pyrazolo­[3,4-*b*]chromene)]-2,5′-dione

**DOI:** 10.1107/S1600536810045253

**Published:** 2010-11-10

**Authors:** Li-Qin Zhao, Bin Li, Yi-Qun Li

**Affiliations:** a Department of Chemistry, Jinan University, Guangzhou 510632, People’s Republic of China

## Abstract

The title spiro­oxindole compound, C_26_H_23_N_3_O_3_, was prepared by the reaction of isatin, 3-methyl-1-phenyl-2-pyrazolin-5-one and 5,5-dimethyl­cyclo­hexane-1,3-dione in an ethanol solution. The fused cyclo­hexene ring adopts an envelope conformation. The dihedral angle between the aromatic and pyrazoline rings is 23.70 (8)°. An intra­molecular C—H⋯O inter­action occurs. The crystal structure is stabilized by N—H⋯N hydrogen-bonding inter­actions, leading to a zigzag chain along the *b* axis.

## Related literature

For general background to spiro compounds and their bio­logical activity, see: Li *et al.* (2010 ▶); Shemchuk *et al.* (2008 ▶); Zhang & Panek (2009 ▶); Zhu *et al.* (2007 ▶).
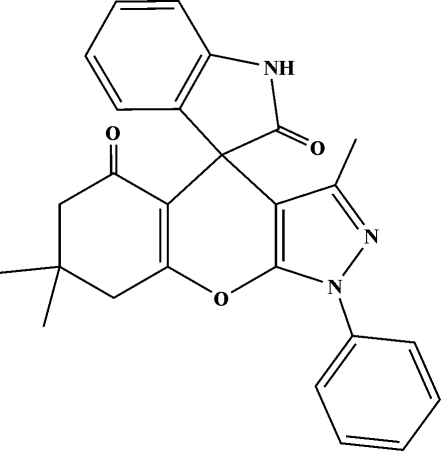


## Experimental

### 

#### Crystal data

C_26_H_23_N_3_O_3_
*M* *_r_* = 425.47
Monoclinic, 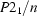

*a* = 11.8778 (19) Å
*b* = 12.891 (2) Å
*c* = 14.039 (2) Å
β = 100.280 (3)°
*V* = 2115.1 (6) Å^3^
*Z* = 4
Mo *K*α radiation
μ = 0.09 mm^−1^
*T* = 110 K
0.46 × 0.40 × 0.39 mm

#### Data collection

Bruker SMART CCD 1K area-detector diffractometer
Absorption correction: multi-scan (*SADABS*; Sheldrick, 1996 ▶) *T*_min_ = 0.960, *T*_max_ = 0.966
9755 measured reflections
4085 independent reflections
3097 reflections with *I* > 2σ(*I*)
*R*_int_ = 0.026

#### Refinement

*R*[*F* ^2^ > 2σ(*F* ^2^)] = 0.039
*wR*(*F* ^2^) = 0.102
*S* = 1.05
4085 reflections
292 parameters
H-atom parameters constrained
Δρ_max_ = 0.26 e Å^−3^
Δρ_min_ = −0.21 e Å^−3^

### 

Data collection: *SMART* (Bruker, 1999 ▶); cell refinement: *SAINT-Plus* (Bruker, 1999 ▶); data reduction: *SAINT-Plus*; program(s) used to solve structure: *SHELXS97* (Sheldrick, 2008 ▶); program(s) used to refine structure: *SHELXL97* (Sheldrick, 2008 ▶); molecular graphics: *SHELXTL* (Sheldrick, 2008 ▶); software used to prepare material for publication: *SHELXTL*.

## Supplementary Material

Crystal structure: contains datablocks I, global. DOI: 10.1107/S1600536810045253/vm2051sup1.cif

Structure factors: contains datablocks I. DOI: 10.1107/S1600536810045253/vm2051Isup2.hkl

Additional supplementary materials:  crystallographic information; 3D view; checkCIF report

## Figures and Tables

**Table 1:** Hydrogen-bond geometry (Å, °)

*D*—H⋯*A*	*D*—H	H⋯*A*	*D*⋯*A*	*D*—H⋯*A*
N3—H3⋯N2^i^	0.88	2.05	2.9185 (18)	172
C12—H12⋯O2	0.95	2.39	2.9780 (19)	119

Symmetry code: (i) 
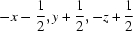
.

**Fig. 1.**
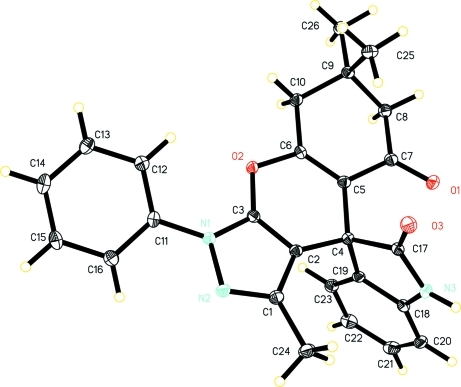
The molecular structure of the title compound in (I) showing the atom numbering scheme. Displacement ellipsoids are drawn at the 50% probability level.

**Fig. 2.**
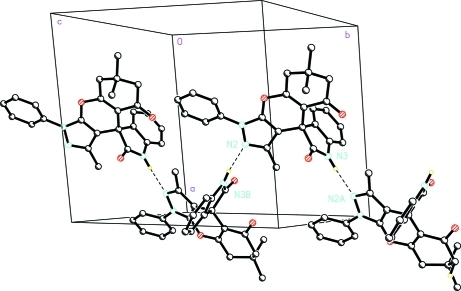
A zigzag hydrogen bonding chain along the b axis is shown in the crystal packing of the title compound. Dashed lines indicate hydrogen bonds. Symmetry operations: A = -x - 1/2, y + 1/2, -z + 1/2; B = -x - 1/2, y - 1/2, -z + 1/2.

## supplementary crystallographic information

### Comment

Spirocyclic systems are probably the most well known heterocycles (Shemchuk *et al.*, 2008). Spiro compounds are characterized by their highly biological activities (Zhu *et al.*, 2007; Li *et al.*, 2010). The spirooxindoles which form the central skeleton of many pharmacologically active compounds and natural products have gained much prominence in recent days (Zhang *et al.*, 2009). Herein, we report the crystal structure of the title compound.

The title compound is a formed from the reaciton of isatin, 3-methyl-1-phenyl-2-pyrazolin-5-one and 5,5-dimethylcyclohexane-1,3-dione. The molecular structure is shown in Fig. 1.

The ring C5—C10 adopts an envelope conformation. The dihedral angle between the aromatic ring C11—C16 and pyrazolin ring (C1—C2—C3—N1—N2) is 23.70 (8) °. The crystal structure is stabilized by N—H···N hydrogen bonding interactions, leading to a zigzag chain along the *b* axis (Fig. 2 and Table 1).

In addition, there is an intramolecular hydrogen bonding interaction between C12 and O2 (Table 1).

### Experimental

The title compound was prepared by the reaction of isatin (0.147 g, 1 mmol), 3-methyl-1-phenyl-2-pyrazolin-5-one (0.174 g, 1 mmol) with 5,5-dimethylcyclohexane-1,3-dione (0.140 g, 1 mmol) in the presence of *p*-TSA (*p*-toluenesulfonic acid) (0.1 g) in water (5.0 ml) under reflux for 24 h. After cooling, the reaction mixture was filtered to collect the solid. The crude product was recrystallized from anhydrous ethanol and then dried to give pure compound (I) in 71% yield (m.p. > 573 K). Single crystals suitable for X-ray diffraction were obtained by slow evaporation of an ethanol solution of (I) at room temperature.

### Refinement

The C-bound H atoms were positioned geometrically and were included in the refinement in the riding-model approximation, with distances 0.98 (CH_3_), 0.99 (CH_2_) and 0.95 Å (aromatic); *U*_iso_(H) = 1.2Ueq(C) for H atoms on secondary and tertiary C atoms, and *U*_iso_ = 1.5Ueq(C) for methyl H atoms. The NH hydrogen atoms were located in a difference Fourier map and then refined as riding on the N atoms with *U*_iso_(H) = 1.2Ueq(N).

### Crystal data

**Table:**

C_26_H_23_N_3_O_3_	*F*(000) = 896
*M**_r_* = 425.47	*D*_x_ = 1.336 Mg m^−^^3^
Monoclinic, *P*2_1_/*n*	Mo *K*α radiation, λ = 0.71073 Å
Hall symbol: -P 2yn	Cell parameters from 4524 reflections
*a* = 11.8778 (19) Å	θ = 2.2–27.0°
*b* = 12.891 (2) Å	µ = 0.09 mm^−^^1^
*c* = 14.039 (2) Å	*T* = 110 K
β = 100.280 (3)°	Block, colorless
*V* = 2115.1 (6) Å^3^	0.46 × 0.40 × 0.39 mm
*Z* = 4	

### Data collection

**Table:**

Bruker SMART CCD 1K area-detector diffractometer	4085 independent reflections
Radiation source: fine-focus sealed tube	3097 reflections with *I* > 2σ(*I*)
graphite	*R*_int_ = 0.026
φ and ω scans	θ_max_ = 26.0°, θ_min_ = 2.1°
Absorption correction: multi-scan (*SADABS*; Sheldrick, 1996)	*h* = −14→14
*T*_min_ = 0.960, *T*_max_ = 0.966	*k* = −15→13
9755 measured reflections	*l* = −17→10

### Refinement

**Table:**

Refinement on *F*^2^	Primary atom site location: structure-invariant direct methods
Least-squares matrix: full	Secondary atom site location: difference Fourier map
*R*[*F*^2^ > 2σ(*F*^2^)] = 0.039	Hydrogen site location: inferred from neighbouring sites
*wR*(*F*^2^) = 0.102	H-atom parameters constrained
*S* = 1.05	*w* = 1/[σ^2^(*F*_o_^2^) + (0.0527*P*)^2^ + 0.4259*P*] where *P* = (*F*_o_^2^ + 2*F*_c_^2^)/3
4085 reflections	(Δ/σ)_max_ < 0.001
292 parameters	Δρ_max_ = 0.26 e Å^−^^3^
0 restraints	Δρ_min_ = −0.20 e Å^−^^3^

### Special details

**Table:**

Geometry. All e.s.d.'s (except the e.s.d. in the dihedral angle between two l.s. planes) are estimated using the full covariance matrix. The cell e.s.d.'s are taken into account individually in the estimation of e.s.d.'s in distances, angles and torsion angles; correlations between e.s.d.'s in cell parameters are only used when they are defined by crystal symmetry. An approximate (isotropic) treatment of cell e.s.d.'s is used for estimating e.s.d.'s involving l.s. planes.
Refinement. Refinement of *F*^2^ against ALL reflections. The weighted *R*-factor *wR* and goodness of fit *S* are based on *F*^2^, conventional *R*-factors *R* are based on *F*, with *F* set to zero for negative *F*^2^. The threshold expression of *F*^2^ > σ(*F*^2^) is used only for calculating *R*-factors(gt) *etc*. and is not relevant to the choice of reflections for refinement. *R*-factors based on *F*^2^ are statistically about twice as large as those based on *F*, and *R*- factors based on ALL data will be even larger.

### Fractional atomic coordinates and isotropic or equivalent isotropic displacement parameters (Å^2^)

**Table:**

	*x*	*y*	*z*	*U*_iso_*/*U*_eq_	
C1	−0.13468 (13)	0.10606 (12)	0.24814 (11)	0.0200 (3)	
C2	−0.04964 (12)	0.17757 (12)	0.23366 (10)	0.0168 (3)	
C3	0.02151 (12)	0.12204 (11)	0.18767 (10)	0.0170 (3)	
C4	−0.02884 (13)	0.28932 (11)	0.26016 (10)	0.0172 (3)	
C5	0.07068 (13)	0.32491 (12)	0.21352 (10)	0.0178 (3)	
C6	0.13557 (13)	0.26188 (11)	0.16884 (10)	0.0179 (3)	
C7	0.09785 (13)	0.43675 (12)	0.21781 (10)	0.0193 (3)	
C8	0.20736 (13)	0.47098 (12)	0.18694 (11)	0.0212 (3)	
H8A	0.2714	0.4627	0.2422	0.025*	
H8B	0.2014	0.5456	0.1700	0.025*	
C9	0.23482 (13)	0.41005 (12)	0.10033 (11)	0.0193 (3)	
C10	0.23633 (13)	0.29460 (12)	0.12630 (11)	0.0207 (3)	
H10A	0.2383	0.2533	0.0672	0.025*	
H10B	0.3071	0.2793	0.1731	0.025*	
C11	0.03029 (13)	−0.06572 (11)	0.13607 (11)	0.0185 (3)	
C12	0.10268 (13)	−0.05500 (12)	0.06905 (11)	0.0205 (3)	
H12	0.1237	0.0120	0.0501	0.025*	
C13	0.14373 (13)	−0.14305 (12)	0.03029 (11)	0.0233 (4)	
H13	0.1927	−0.1364	−0.0160	0.028*	
C14	0.11399 (14)	−0.24068 (13)	0.05836 (12)	0.0275 (4)	
H14	0.1416	−0.3008	0.0308	0.033*	
C15	0.04423 (15)	−0.25055 (13)	0.12654 (12)	0.0267 (4)	
H15	0.0254	−0.3176	0.1469	0.032*	
C16	0.00131 (14)	−0.16334 (12)	0.16572 (11)	0.0224 (4)	
H16	−0.0473	−0.1704	0.2122	0.027*	
C17	−0.14039 (13)	0.35270 (11)	0.22569 (11)	0.0192 (3)	
C18	−0.10051 (13)	0.36495 (11)	0.39106 (11)	0.0204 (3)	
C19	−0.01059 (13)	0.30626 (11)	0.36897 (10)	0.0180 (3)	
C20	−0.10638 (15)	0.39146 (13)	0.48573 (12)	0.0259 (4)	
H20	−0.1673	0.4327	0.5005	0.031*	
C21	−0.01995 (15)	0.35545 (13)	0.55838 (12)	0.0287 (4)	
H21	−0.0214	0.3731	0.6238	0.034*	
C22	0.06789 (15)	0.29442 (13)	0.53702 (12)	0.0282 (4)	
H22	0.1250	0.2694	0.5879	0.034*	
C23	0.07337 (13)	0.26930 (12)	0.44129 (11)	0.0226 (4)	
H23	0.1338	0.2276	0.4264	0.027*	
C24	−0.23692 (14)	0.12431 (13)	0.29448 (12)	0.0267 (4)	
H24A	−0.2972	0.1581	0.2480	0.040*	
H24B	−0.2155	0.1690	0.3513	0.040*	
H24C	−0.2651	0.0578	0.3145	0.040*	
C25	0.14278 (14)	0.43141 (13)	0.01188 (11)	0.0247 (4)	
H25A	0.1557	0.3871	−0.0418	0.037*	
H25B	0.1463	0.5044	−0.0069	0.037*	
H25C	0.0673	0.4165	0.0276	0.037*	
C26	0.35231 (14)	0.44152 (13)	0.07888 (12)	0.0252 (4)	
H26A	0.4105	0.4318	0.1371	0.038*	
H26B	0.3505	0.5146	0.0594	0.038*	
H26C	0.3710	0.3983	0.0264	0.038*	
N1	−0.01647 (11)	0.02305 (10)	0.17519 (9)	0.0180 (3)	
N2	−0.11516 (11)	0.01329 (10)	0.21348 (9)	0.0205 (3)	
N3	−0.17731 (11)	0.38803 (10)	0.30610 (9)	0.0227 (3)	
H3	−0.2421	0.4216	0.3048	0.027*	
O1	0.03312 (10)	0.49811 (8)	0.24678 (8)	0.0264 (3)	
O2	0.11714 (9)	0.15614 (8)	0.15647 (7)	0.0199 (2)	
O3	−0.18860 (9)	0.36251 (9)	0.14228 (8)	0.0260 (3)	

### Atomic displacement parameters (Å^2^)

**Table:**

	*U*^11^	*U*^22^	*U*^33^	*U*^12^	*U*^13^	*U*^23^
C1	0.0206 (8)	0.0224 (8)	0.0170 (7)	−0.0002 (7)	0.0031 (6)	−0.0006 (6)
C2	0.0164 (8)	0.0186 (8)	0.0155 (7)	0.0007 (6)	0.0030 (6)	0.0011 (6)
C3	0.0156 (7)	0.0172 (8)	0.0181 (7)	0.0002 (6)	0.0030 (6)	0.0016 (6)
C4	0.0166 (8)	0.0167 (8)	0.0188 (8)	0.0004 (6)	0.0048 (6)	0.0001 (6)
C5	0.0171 (8)	0.0196 (8)	0.0166 (7)	0.0000 (6)	0.0025 (6)	0.0013 (6)
C6	0.0189 (8)	0.0148 (8)	0.0195 (8)	−0.0007 (6)	0.0020 (6)	0.0009 (6)
C7	0.0233 (8)	0.0206 (8)	0.0136 (7)	0.0000 (7)	0.0020 (6)	−0.0005 (6)
C8	0.0224 (8)	0.0192 (8)	0.0221 (8)	−0.0031 (6)	0.0040 (7)	−0.0023 (6)
C9	0.0195 (8)	0.0183 (8)	0.0213 (8)	−0.0028 (6)	0.0067 (6)	−0.0003 (6)
C10	0.0178 (8)	0.0205 (8)	0.0251 (8)	−0.0006 (6)	0.0074 (7)	−0.0012 (6)
C11	0.0170 (7)	0.0175 (8)	0.0190 (8)	0.0021 (6)	−0.0018 (6)	−0.0031 (6)
C12	0.0200 (8)	0.0215 (8)	0.0189 (8)	0.0002 (6)	0.0002 (6)	0.0006 (6)
C13	0.0190 (8)	0.0273 (9)	0.0229 (8)	0.0028 (7)	0.0018 (7)	−0.0044 (7)
C14	0.0248 (9)	0.0239 (9)	0.0318 (9)	0.0049 (7)	−0.0002 (7)	−0.0088 (7)
C15	0.0281 (9)	0.0173 (8)	0.0327 (9)	−0.0021 (7)	0.0002 (7)	−0.0021 (7)
C16	0.0216 (8)	0.0215 (8)	0.0230 (8)	−0.0017 (7)	0.0013 (7)	0.0001 (6)
C17	0.0188 (8)	0.0153 (8)	0.0246 (8)	−0.0004 (6)	0.0070 (7)	0.0024 (6)
C18	0.0227 (8)	0.0156 (8)	0.0243 (8)	−0.0018 (6)	0.0079 (7)	−0.0001 (6)
C19	0.0196 (8)	0.0159 (8)	0.0198 (8)	−0.0045 (6)	0.0075 (6)	−0.0021 (6)
C20	0.0320 (10)	0.0197 (8)	0.0304 (9)	−0.0023 (7)	0.0171 (8)	−0.0052 (7)
C21	0.0369 (10)	0.0311 (10)	0.0200 (8)	−0.0106 (8)	0.0100 (7)	−0.0073 (7)
C22	0.0299 (10)	0.0317 (10)	0.0218 (8)	−0.0067 (8)	0.0014 (7)	−0.0010 (7)
C23	0.0195 (8)	0.0234 (9)	0.0250 (8)	−0.0017 (7)	0.0045 (7)	−0.0005 (6)
C24	0.0255 (9)	0.0275 (9)	0.0302 (9)	−0.0040 (7)	0.0129 (7)	−0.0043 (7)
C25	0.0233 (8)	0.0303 (9)	0.0213 (8)	−0.0005 (7)	0.0061 (7)	0.0018 (7)
C26	0.0226 (9)	0.0253 (9)	0.0292 (9)	−0.0033 (7)	0.0086 (7)	−0.0011 (7)
N1	0.0176 (7)	0.0170 (7)	0.0206 (7)	−0.0008 (5)	0.0063 (5)	−0.0005 (5)
N2	0.0193 (7)	0.0216 (7)	0.0219 (7)	−0.0026 (5)	0.0075 (5)	−0.0016 (5)
N3	0.0204 (7)	0.0217 (7)	0.0280 (7)	0.0065 (6)	0.0095 (6)	0.0025 (6)
O1	0.0321 (7)	0.0195 (6)	0.0309 (6)	0.0012 (5)	0.0145 (5)	−0.0034 (5)
O2	0.0178 (6)	0.0154 (5)	0.0284 (6)	−0.0006 (4)	0.0095 (5)	−0.0013 (4)
O3	0.0247 (6)	0.0285 (7)	0.0237 (6)	0.0039 (5)	0.0017 (5)	0.0043 (5)

### Geometric parameters (Å, °)

**Table:**

C1—N2	1.327 (2)	C13—H13	0.9500
C1—C2	1.409 (2)	C14—C15	1.379 (2)
C1—C24	1.495 (2)	C14—H14	0.9500
C2—C3	1.356 (2)	C15—C16	1.388 (2)
C2—C4	1.497 (2)	C15—H15	0.9500
C3—N1	1.3543 (19)	C16—H16	0.9500
C3—O2	1.3612 (17)	C17—O3	1.2148 (18)
C4—C19	1.520 (2)	C17—N3	1.3606 (19)
C4—C5	1.521 (2)	C18—C20	1.386 (2)
C4—C17	1.558 (2)	C18—C19	1.388 (2)
C5—C6	1.349 (2)	C18—N3	1.398 (2)
C5—C7	1.476 (2)	C19—C23	1.375 (2)
C6—O2	1.3866 (18)	C20—C21	1.391 (2)
C6—C10	1.491 (2)	C20—H20	0.9500
C7—O1	1.2223 (18)	C21—C22	1.382 (2)
C7—C8	1.509 (2)	C21—H21	0.9500
C8—C9	1.531 (2)	C22—C23	1.395 (2)
C8—H8A	0.9900	C22—H22	0.9500
C8—H8B	0.9900	C23—H23	0.9500
C9—C25	1.526 (2)	C24—H24A	0.9800
C9—C10	1.532 (2)	C24—H24B	0.9800
C9—C26	1.534 (2)	C24—H24C	0.9800
C10—H10A	0.9900	C25—H25A	0.9800
C10—H10B	0.9900	C25—H25B	0.9800
C11—C16	1.388 (2)	C25—H25C	0.9800
C11—C12	1.390 (2)	C26—H26A	0.9800
C11—N1	1.4243 (19)	C26—H26B	0.9800
C12—C13	1.385 (2)	C26—H26C	0.9800
C12—H12	0.9500	N1—N2	1.3798 (17)
C13—C14	1.383 (2)	N3—H3	0.8800
			
N2—C1—C2	111.11 (13)	C14—C15—C16	120.59 (15)
N2—C1—C24	120.66 (14)	C14—C15—H15	119.7
C2—C1—C24	128.23 (14)	C16—C15—H15	119.7
C3—C2—C1	104.30 (13)	C11—C16—C15	119.16 (15)
C3—C2—C4	122.48 (13)	C11—C16—H16	120.4
C1—C2—C4	133.20 (13)	C15—C16—H16	120.4
N1—C3—C2	109.73 (13)	O3—C17—N3	126.78 (14)
N1—C3—O2	122.72 (13)	O3—C17—C4	125.62 (13)
C2—C3—O2	127.55 (14)	N3—C17—C4	107.45 (12)
C2—C4—C19	112.06 (12)	C20—C18—C19	121.39 (15)
C2—C4—C5	106.84 (12)	C20—C18—N3	128.96 (15)
C19—C4—C5	114.02 (12)	C19—C18—N3	109.64 (13)
C2—C4—C17	109.50 (12)	C23—C19—C18	120.61 (14)
C19—C4—C17	101.41 (12)	C23—C19—C4	130.31 (14)
C5—C4—C17	113.03 (12)	C18—C19—C4	108.99 (13)
C6—C5—C7	117.92 (14)	C18—C20—C21	117.64 (15)
C6—C5—C4	124.82 (14)	C18—C20—H20	121.2
C7—C5—C4	117.27 (13)	C21—C20—H20	121.2
C5—C6—O2	124.03 (13)	C22—C21—C20	121.19 (15)
C5—C6—C10	125.59 (14)	C22—C21—H21	119.4
O2—C6—C10	110.37 (12)	C20—C21—H21	119.4
O1—C7—C5	119.98 (14)	C21—C22—C23	120.53 (16)
O1—C7—C8	122.15 (14)	C21—C22—H22	119.7
C5—C7—C8	117.87 (13)	C23—C22—H22	119.7
C7—C8—C9	113.26 (12)	C19—C23—C22	118.59 (15)
C7—C8—H8A	108.9	C19—C23—H23	120.7
C9—C8—H8A	108.9	C22—C23—H23	120.7
C7—C8—H8B	108.9	C1—C24—H24A	109.5
C9—C8—H8B	108.9	C1—C24—H24B	109.5
H8A—C8—H8B	107.7	H24A—C24—H24B	109.5
C25—C9—C8	109.30 (13)	C1—C24—H24C	109.5
C25—C9—C10	110.20 (13)	H24A—C24—H24C	109.5
C8—C9—C10	107.73 (12)	H24B—C24—H24C	109.5
C25—C9—C26	109.71 (13)	C9—C25—H25A	109.5
C8—C9—C26	110.52 (13)	C9—C25—H25B	109.5
C10—C9—C26	109.35 (13)	H25A—C25—H25B	109.5
C6—C10—C9	113.21 (13)	C9—C25—H25C	109.5
C6—C10—H10A	108.9	H25A—C25—H25C	109.5
C9—C10—H10A	108.9	H25B—C25—H25C	109.5
C6—C10—H10B	108.9	C9—C26—H26A	109.5
C9—C10—H10B	108.9	C9—C26—H26B	109.5
H10A—C10—H10B	107.7	H26A—C26—H26B	109.5
C16—C11—C12	120.63 (14)	C9—C26—H26C	109.5
C16—C11—N1	118.57 (14)	H26A—C26—H26C	109.5
C12—C11—N1	120.79 (14)	H26B—C26—H26C	109.5
C13—C12—C11	119.23 (15)	C3—N1—N2	108.79 (12)
C13—C12—H12	120.4	C3—N1—C11	131.54 (13)
C11—C12—H12	120.4	N2—N1—C11	119.59 (12)
C14—C13—C12	120.54 (15)	C1—N2—N1	106.07 (12)
C14—C13—H13	119.7	C17—N3—C18	112.26 (13)
C12—C13—H13	119.7	C17—N3—H3	123.9
C15—C14—C13	119.82 (15)	C18—N3—H3	123.9
C15—C14—H14	120.1	C3—O2—C6	113.54 (11)
C13—C14—H14	120.1		
			
N2—C1—C2—C3	0.37 (17)	C14—C15—C16—C11	−0.6 (2)
C24—C1—C2—C3	−179.09 (15)	C2—C4—C17—O3	−61.79 (19)
N2—C1—C2—C4	−178.23 (15)	C19—C4—C17—O3	179.66 (14)
C24—C1—C2—C4	2.3 (3)	C5—C4—C17—O3	57.2 (2)
C1—C2—C3—N1	−0.38 (16)	C2—C4—C17—N3	113.94 (13)
C4—C2—C3—N1	178.42 (13)	C19—C4—C17—N3	−4.61 (15)
C1—C2—C3—O2	179.22 (14)	C5—C4—C17—N3	−127.08 (13)
C4—C2—C3—O2	−2.0 (2)	C20—C18—C19—C23	2.5 (2)
C3—C2—C4—C19	−117.71 (15)	N3—C18—C19—C23	−176.67 (13)
C1—C2—C4—C19	60.7 (2)	C20—C18—C19—C4	179.44 (14)
C3—C2—C4—C5	7.85 (19)	N3—C18—C19—C4	0.28 (17)
C1—C2—C4—C5	−173.75 (15)	C2—C4—C19—C23	62.4 (2)
C3—C2—C4—C17	130.58 (15)	C5—C4—C19—C23	−59.1 (2)
C1—C2—C4—C17	−51.0 (2)	C17—C4—C19—C23	179.12 (15)
C2—C4—C5—C6	−8.21 (19)	C2—C4—C19—C18	−114.12 (14)
C19—C4—C5—C6	116.16 (16)	C5—C4—C19—C18	124.35 (14)
C17—C4—C5—C6	−128.71 (15)	C17—C4—C19—C18	2.56 (15)
C2—C4—C5—C7	171.85 (12)	C19—C18—C20—C21	−1.2 (2)
C19—C4—C5—C7	−63.78 (17)	N3—C18—C20—C21	177.76 (15)
C17—C4—C5—C7	51.35 (17)	C18—C20—C21—C22	−0.7 (2)
C7—C5—C6—O2	−177.59 (13)	C20—C21—C22—C23	1.5 (3)
C4—C5—C6—O2	2.5 (2)	C18—C19—C23—C22	−1.7 (2)
C7—C5—C6—C10	2.6 (2)	C4—C19—C23—C22	−177.94 (15)
C4—C5—C6—C10	−177.33 (14)	C21—C22—C23—C19	−0.2 (2)
C6—C5—C7—O1	171.01 (14)	C2—C3—N1—N2	0.26 (16)
C4—C5—C7—O1	−9.1 (2)	O2—C3—N1—N2	−179.36 (13)
C6—C5—C7—C8	−9.8 (2)	C2—C3—N1—C11	−176.30 (14)
C4—C5—C7—C8	170.11 (13)	O2—C3—N1—C11	4.1 (2)
O1—C7—C8—C9	−143.11 (15)	C16—C11—N1—C3	154.44 (15)
C5—C7—C8—C9	37.76 (19)	C12—C11—N1—C3	−26.1 (2)
C7—C8—C9—C25	64.51 (17)	C16—C11—N1—N2	−21.8 (2)
C7—C8—C9—C10	−55.24 (17)	C12—C11—N1—N2	157.68 (13)
C7—C8—C9—C26	−174.65 (13)	C2—C1—N2—N1	−0.22 (16)
C5—C6—C10—C9	−23.1 (2)	C24—C1—N2—N1	179.28 (13)
O2—C6—C10—C9	157.05 (12)	C3—N1—N2—C1	−0.02 (16)
C25—C9—C10—C6	−71.83 (16)	C11—N1—N2—C1	177.02 (12)
C8—C9—C10—C6	47.34 (17)	O3—C17—N3—C18	−179.12 (15)
C26—C9—C10—C6	167.49 (13)	C4—C17—N3—C18	5.21 (16)
C16—C11—C12—C13	1.5 (2)	C20—C18—N3—C17	177.31 (15)
N1—C11—C12—C13	−178.02 (13)	C19—C18—N3—C17	−3.61 (18)
C11—C12—C13—C14	−0.5 (2)	N1—C3—O2—C6	174.73 (13)
C12—C13—C14—C15	−0.9 (2)	C2—C3—O2—C6	−4.8 (2)
C13—C14—C15—C16	1.5 (2)	C5—C6—O2—C3	4.5 (2)
C12—C11—C16—C15	−0.9 (2)	C10—C6—O2—C3	−175.68 (12)
N1—C11—C16—C15	178.57 (14)		

### Hydrogen-bond geometry (Å, °)

**Table:**

*D*—H···*A*	*D*—H	H···*A*	*D*···*A*	*D*—H···*A*
N3—H3···N2^i^	0.88	2.05	2.9185 (18)	172
C12—H12···O2	0.95	2.39	2.9780 (19)	119

Symmetry codes: (i) −*x*−1/2, *y*+1/2, −*z*+1/2.
